# Acupuncture for the treatment of diarrheal-predominant irritable bowel syndrome: study protocol for a pilot randomized controlled trial

**DOI:** 10.1186/s13063-021-05211-x

**Published:** 2021-04-07

**Authors:** Ling-Yu Qi, Yu Wang, Li-Qiong Wang, Yan-Fen She, Guang-Xia Shi, Ying Li, Li-Li Chi, Bang-Qi Wu, Jian-Feng Tu, Ying Lin, Fang-Ting Yu, Jing-Wen Yang, Cun-Zhi Liu

**Affiliations:** 1 International Acupuncture and Moxibustion Innovation Institute, School of Acupuncture-Moxibustion and Tuina, Beijing University of Chinese Medicine, 100029 Beijing, China; 2 School of Acupuncture-Moxibustion and Tuina, Hebei University of Chinese Medicine, Shijiazhuang, 050299 China; 3 School of Graduate, Chengdu University of Chinese Medicine, Chengdu, 610075 China; 4 Department of Spleen and Stomach, the Affiliated Hospital of Shandong University of Traditional Chinese Medicine, Jinan, 250011 China; 5 National Acupuncture and Moxibustion Clinical Medical Research Center, the First Teaching Hospital of Tianjin University of Traditional Chinese Medicine, Tianjin, 300193 China

**Keywords:** Irritable bowel syndrome, Acupuncture, Specific acupoints, Randomized controlled trial

## Abstract

**Background:**

Irritable bowel syndrome (IBS) is one of the most common functional gastrointestinal diseases. Although acupuncture has become a common alternative therapy for IBS, there is insufficient evidence for its effectiveness. This study was designed to assess the efficacy and feasibility of acupuncture in the treatment of IBS.

**Methods/design:**

This is a multicenter randomized controlled clinical trial. According to the ratio of 1:1:1, 90 patients with irritable bowel syndrome will be randomly divided into specific acupoints (SA) group, non-specific acupoints (NSA) group, and non-acupoints (NA) group. All patients will be treated with acupuncture 12 times within 4 weeks and followed up for 8 weeks. The primary outcome is the response rate, the percentage of patients whose average value of worst abdominal pain is 30% better and the days of loose stool is 50% less than the baseline, at week 4 after randomization. The secondary outcomes include the response rates at other time points, IBS Symptom Severity Scale (IBS-SSS), Patient Health Questionnaire-9 depression scale (PHQ-9), IBS-Quality of Life scale (IBS-QOL), IBS Adequate Relief (IBS-AR), Abdominal Pain Score, Abdominal Bloating Score, Bristol Stool Score (BBS), blinding assessment, and credibility evaluation. Adverse events will be monitored and recorded during the trial.

**Trial registration:**

Chictr.org.cn ChiCTR2000030670. Registered on 9 March 2020.

## Background and objectives

Irritable bowel syndrome (IBS) is a functional gastrointestinal disease [1], with abdominal pain, abdominal distension, changes in defecation habits, and stool irregularities as the main clinical manifestations [2]. The global prevalence of IBS is approximately 11% [3], while the prevalence in different regions of Asia ranges from 5.0% to 9.9% [4]. According to the Rome IV criteria [1], IBS were subgrouped into diarrheal-predominant IBS (IBS-D), constipation-predominant IBS (IBS-C), mixed IBS (IBS-M), and IBS unclassifable (IBS-U), among which IBS-D is the most frequently occurring subtype accounting for 40% [4]. Furthermore, IBS, one of the most common reasons for the abnormal state of life and work [5], increased the economic burden and impaired the life quality of patients [6, 7].

The pathophysiology of IBS is still poorly elucidated, thereby hampering the development of effective therapies for IBS [8]. Currently, available IBS treatments were broadly classified into drug therapies and other interventions, such as lifestyle and dietary modifications [9, 10]. Most IBS drugs are limited to the symptomatic treatment of specific symptoms of IBS which has no significant effectiveness on other related symptoms [11]. And lifestyle and dietary modifications were not uniformly effective across all patients. In this context, complementary and alternative medicine has shown promise [12]. As one of the treatments in complementary and alternative medicine, acupuncture is considered a beneficial alternative treatment for functional gastrointestinal disorders [13].

Acupuncture has been applied to gastrointestinal diseases in China for thousands of years, and its therapeutic effectiveness in IBS is being increasingly approved by more individuals [14]. Acupuncture is believed to alter visceral sensation and motility by interfering with the relevant mediators of the somatic nervous system and the central nervous system [15, 16]. In addition, studies have also shown that acupuncture can interfere with IBS from aspects of the brain-gut axis [17, 18] and the immune system [19]. However, the studies to date provided insufficient evidence to determine whether acupuncture is an effective treatment for IBS. A previous randomized controlled study assigned patients to receive either acupuncture (*n* = 22) or sham acupuncture (*n* = 21) concluded that “acupuncture in IBS is primarily a placebo response” [20]. But a total of 10 acupuncture/sham acupuncture sessions and sample size of 43 were considered insufficient, which may increase the likelihood of negative results. Furthermore, the acupoint is the key factor to determine the curative effect of acupuncture. Specific acupoints refer to the acupoints with special therapeutic function and naming in the meridians, which have specific treatment rules and wide applications. Comparing the therapeutic effects of different acupoint selection schemes is helpful to optimize the acupoint selection scheme and improve the clinical efficacy of acupuncture treatment, while the clinical efficacy among specific acupoints (SA), non-specific acupoints (NSA), and non-acupoints (NA) have not been revealed.

Therefore, we designed this clinical trial with the following two objectives: (I) explore the efficacy and safety of acupuncture in the treatment of IBS to guide the implementation of the large-scale trial; and (II) compare the different therapeutic effects of acupoint acupuncture groups and sham acupuncture group.

## Methods

### Trial design

**Fig. 1:**
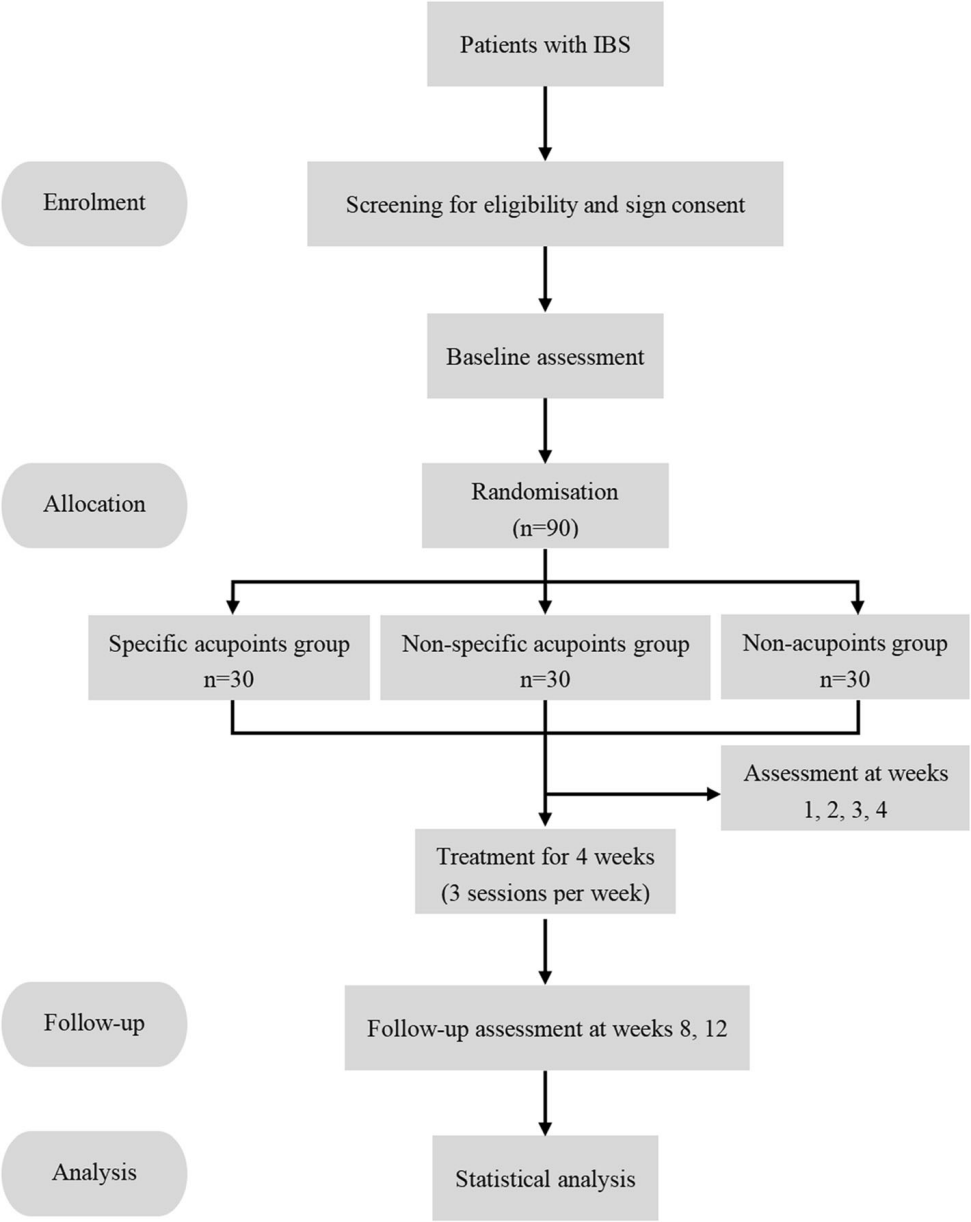
The flow diagram of this trial

This is a study protocol for a randomized, multicenter, clinical trial. Patients with IBS will be randomly assigned to the SA group, the NSA group, or the NA group in a 1:1:1 ratio (Fig. 1).

### Participants

This trial is an exploratory trial which will be conducted in four hospitals in China, including: (I) The Affiliated Hospital of Hebei University of Chinese Medicine; (II) the First Teaching Hospital of Tianjin University of Traditional Chinese Medicine; (III) the Teaching Hospital of Chengdu University of Traditional Chinese Medicine; and (IV) the Affiliated Hospital of Shandong University of Traditional Chinese Medicine. Patients who meet the IBS criteria of Rome IV will be recruited mainly through outpatient clinics, advertisements on hospital social media and newspapers. All patients will receive and fill in the informed consent before randomization.

#### Inclusion criteria

(I) Aged between 18 and 75 years (either sex);
(II) Diarrheal-predominant irritable bowel syndrome (IBS-D); and
(III) Type 6 or 7 of the Bristol Stool Form Scale appeared for at least 4 days and type 1 or 2 appeared for less than 4 days in the last 2 weeks; the average score of daily abdominal pain was ≥ 3 in the last week.

#### Exclusion criteria

(I) Patients with the following diseases: inflammatory bowel disease, microscopic colitis, celiac disease, Crohn’s disease, and other organic bowel diseases; diabetes mellitus and abnormal thyroid function; severe acute or chronic organic diseases, kidney or liver diseases;
(II) History of previous abdominal surgery (appendectomy, hemorrhoidectomy, or polypectomy greater than 3 months post-surgery are allowed);
(III) Pregnancy or lactation or history of alcohol and drug abuse;
(IV) Treated with acupuncture in the last 6 months or participating in other clinical trials; and
(V) Usage of antidepressant or IBS medication within 2 weeks before treatment, including traditional Chinese medicine (TCM) or proprietary Chinese medicine, antidiarrheal, antispasmodic, intestinal antibiotics, probiotics, and so on.

### Interventions

Before acupuncture treatment, licensed acupuncturists with at least 3 years of acupuncture experience will be trained in how to locate acupoints and non-acupoints, puncture and manipulate needles. All acupoints are localized according to the WHO Standard. Single-use sterile needles (0.30 mm in diameter and 30 mm in length or 0.30 mm in diameter and 25 mm in length; Hwato, Suzhou, China) will be used in the SA group and NSA group. To help maximize blinding of patients in the NA group, a blunt-tipped placebo needle will be used (similar to the Streitberger design [21]) which can provide patient-blinding effects with a similar appearance to conventional needles but no skin penetration [22]. And adhesive pads will be used in each group. Each patient will be scheduled to undergo 12 sessions of treatment, with each acupuncture lasting 30 min for 4 weeks (three sessions per week, every other day ideally). During the trial, patients will not be allowed to use other therapies or medications that have a therapeutic effect on IBS, such as traditional Chinese medicine (TCM) or proprietary Chinese medicine, antidiarrheal, antispasmodic, intestinal antibiotics, probiotics, and so on. Loperamide (Imodium, Xian Janssen Pharmaceutical Ltd., China) will be used as rescue medication under the guidance of gastroenterologists whenever necessary. The medication status and other non-irritable bowel syndrome drug applications of patients will be strictly recorded during the trial.

#### Specific acupoints group

The acupoints used in this group are composed of fixed acupoints and optional acupoints. Fixed acupoints include *Tianshu* (ST25), *Zhongwan* (RN12), *Guanyuan* (CV4), *Zusanli* (ST36), and *Shangjuxu* (ST37), which are commonly used in IBS patients. Optional acupoints will be selected based on the traditional Chinese acupuncture diagnosis of patients by the acupuncturist, such as *Taichong* (LR3) for syndrome of liver depression and spleen deficiency, *Sanyinjiao* (SP6) for syndrome of spleen deficiency and dampness obstruction, and *Neiting* (ST44) for syndrome of spleen-stomachdamp-heat. After sterilization, the acupuncturist will insert single-use sterile needles into the deep tissue layers through adhesive pads of acupoints. Following needle insertion, small, equal manipulations of twirling, lifting, and thrusting will be performed on all needles to reach *de qi* (a component sensation, including soreness, numbness, distension, and heaviness). Acupuncture Locations and exhibited in Table 1.

**Table 1:** Locations of acupoints for SA group

	Acupoint	Location
Fixed acupoints of SA group	*Tianshu* (ST25)	On the horizontal line of the navel, 2 cun^a^ beside the anterior midline
	*Zhongwan* (RN12)	On the anterior midline of the upper abdomen, 4 cun superior to the navel
	*Guanyuan* (CV4)	On the anterior midline of the abdomen, 3 cun inferior to the navel
	*Zusanli* (ST36)	3 cun directly below ST35, and one finger-breadth lateral to the anterior border of the tibia
	*Shangjuxu* (ST37)	On the anterolateral aspect of the leg, 6 cun inferior to the ST35, and one finger-breadth lateral to the anterior border of the tibia
Optional acupoints of SA group	*Taichong* (LR3)	In the depression anterior to the junction of the first and second metatarsal bones
	*Sanyinjiao* (SP6)	On the tibial aspect of the leg, posterior to the medial border of the tibia, 3 cun superior to the prominence of the medial malleolus
	*Neiting* (ST44)	On the instep, between the second and third toes of the red and white flesh behind the webbed margin

^a^1 cun (≈20 mm) is defined as the width of the interphalangeal joint of the patient’s thumb

#### Non-specific acupoints group

The acupoints used in this group consist of six fixed acupoints (Table 2): *Shuifen* (RN9), *Liangmen* (ST21), *Yinjiao* (CV7), *Tiaokou* (ST38), *Yinshi* (ST33), *Lougu* (SP7), *Yinbao* (LR9). The rest of the operation is the same as that of the SA group.

**Table 2:** Locations of acupoints for NSA group

	Acupoint	Location
Fixed acupoints of NSA group	*Shuifen* (RN9)	On the anterior midline of the upper abdomen, 1 cun^a^ superior to the navel
	*Liangmen* (ST21)	On the anterior midline of the upper abdomen, 1 cun superior to the navel, 2 cun beside the anterior midline
	*Yinjiao* (CV7)	On the anterior midline of the abdomen, 1 cun inferior to the navel
	*Tiaokou* (ST38)	On the anterolateral aspect of the leg, 8 cun inferior to the ST35, and on the line between ST35 and ST41
	*Yinshi* (ST33)	On the anterior aspect of the thigh, 3 cun superior to basis patellae, and on the line between anterior superior iliac spine and basis patellae
	*Lougu* (SP7)	On the anteromedial aspect of the leg and the tibial rear, on the line between medial malleolus and SP9, and 6 cun superior to medial malleolus

^a^1 cun (≈20 mm) is defined as the width of the interphalangeal joint of the patient’s thumb

#### Non-acupoints group

Patients in this group will receive sham acupuncture with blunt-tipped placebo needles on non-acupoints. Five non-acupoints will be selected which are away from meridians or conventional acupoints. The use of blunt-tipped placebo needles will provide patients with the feeling of acupuncture but with no skin penetration and needle manipulation for *de qi*. The non-acupoints are shown in Table 3.

**Table 3:** Locations of acupoints for NA group

NA	Location
NA1	On the abdomen, 2 cun^a^ superior to anterior superior iliac spine, between the gallbladder meridian and the spleen meridian
NA2	On the abdomen, 2 cun inferior to navel, 1 cun beside the anterior midline, between the kidney meridian and the stomach meridian
NA3	On the lateral aspect of the leg, 3 cun inferior to GB34, between gallbladder meridian and bladder meridian
NA4	On the leg, 2 cun superior to the medial malleolus, in the middle of the medial tibia, between the liver meridian and the spleen meridian
NA5	On the leg, the midpoint of the line between GB40 and ST41, between the gallbladder meridian and the stomach meridian

^a^1 cun (≈20 mm) is defined as the width of the interphalangeal joint of the patient’s thumb

### Outcomes

#### Primary outcome

In this trial, the response rate defined as the percentage of patients whose average value of worst abdominal pain is 30% better and the days of loose stool is 50% less than the baseline. The primary outcome is the response rate of week 4 after randomization. Patients will be asked to record daily IBS symptoms, including worst abdominal pain and stool type through Bristol Stool Scale. The score of worst abdominal pain in the preceding 24 h will be evaluated by visual analog scale (VAS), and the average value of worst abdominal pain will be calculated weekly.

#### Secondary outcomes

##### The response rates at other time points

The response rate will also be measured at weeks 1, 2, 3, 8, and 12 after randomization.

##### IBS Symptom Severity Scale (IBS-SSS)

The IBS-SSS contains five questions that are rated on VAS (0–100): severity of abdominal pain, frequency of abdominal pain, severity of abdominal distension, degree of dissatisfaction with defecation habits, and interference with the quality of life. The above five aspects account for an equal proportion in the IBS-SSS with a score range of 0–500. A score below 175 indicates that the mild IBS syndrome, a score between 175 and 300, represents the moderate IBS syndrome and a score higher than 300 represents the severe IBS syndrome [23]. IBS-SSS will be used at weeks 2, 4, 8, and 12 after randomization.

##### Patient Health Questionnaire-9 depression scale (PHQ-9)

PHQ-9 scores range from 0 to 27, by which depression is defined as mild (5–9), moderate (10–14), moderate (15–19), or severe (more than 20) [24]. PHQ-9 will be used at weeks 2, 4, 8, and 12 after randomization.

##### IBS-Quality of Life scale (IBS-QOL)

IBS-QOL, which consists of 34 items, will be used to assess the extent to which the quality of life of patients with IBS is disturbed. Each item will be evaluated with a 5-point Likert scale, and the cumulative total score of all items will be linearly converted into a 100-point scale. The higher score indicates the improvement in quality of life is more obvious [25]. IBS-QOL will be used at weeks 2, 4, 8, and 12 after randomization.

##### IBS Adequate Relief (IBS-AR)

IBS-AR will be used to confirm whether the IBS symptoms of trial patients have been adequately relieved. This type of outcome has been shown to be associated with the improvement of individual symptoms [26] and has been extensively used to evaluate efficacy in IBS clinical trials [27, 28]. The responder is defined as the patient who answered the question in the affirmative. IBS-AR will be used at weeks 1, 2, 3, 4, 8, and 12 after randomization.

##### Abdominal Pain Score

Patients will be asked to rate their worst abdominal pain in the past 24 h. The pain will be recorded on VAS (0–10), where 0 corresponds to no pain and 10 corresponds to worst imaginable pain. The score will be measured at weeks 1, 2, 3, 4, 8, and 12 after randomization.

##### Abdominal Bloating Score

Patients will be asked to rate their worst abdominal bloating in the past 24 h. The bloating will be recorded on VAS (0–10), where 0 corresponds to no bloating and 10 corresponds to worst imaginable bloating. The score will be measured at weeks 1, 2, 3, 4, 8, and 12 after randomization.

##### Bristol Stool Score (BBS)

Patients will be asked to rate the BBS of the past 24 h. The score is based on a 1 to 7 scale where 1 corresponds to a hard stool and 7 corresponds to watery diarrhea. And the frequency of each score will be also recorded. The BBS will be measured at weeks 1, 2, 3, 4, 8, and 12 after randomization.

##### Blinding assessment

To test whether blinding is successful, all patients will be asked to guess which kind of acupuncture they received at week 4 after randomization.

##### Credibility and expectancy

Credibility and expectancy will be evaluated using the Credibility/Expectancy Questionnaire within 5 min after the first treatment.

**Fig. 2:**
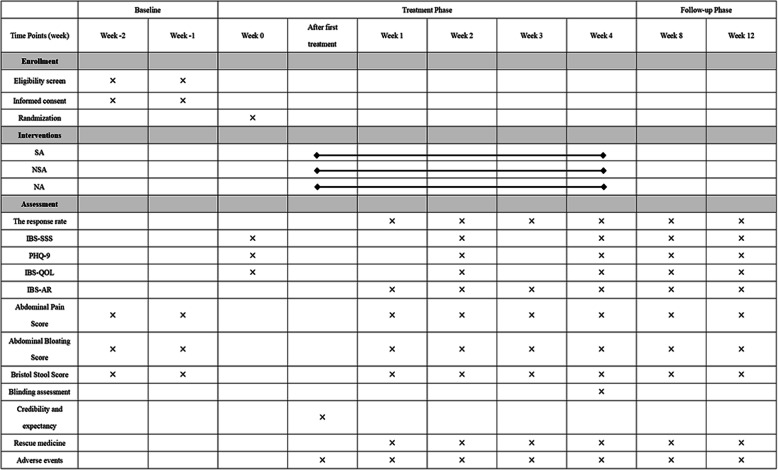
Schedule of enrollment, intervention, and assessments

The schedule of enrollment, intervention, and assessments is shown in Fig. 2.

### Sample size

This pilot study aimed to assess the effectiveness of different acupuncture intervention for IBS and determine the feasibility of a further large clinical trial. The minimum sample size for exploratory trials is 20–30 per group according to Provisions for Drug Registration in China. We selected the maximum of 30 patients, and the sample size of 90 patients was determined. The results of this study will facilitate the calculation of an appropriate sample size for further randomized clinical trials.

### Randomization

#### Sequence generation

Patients need to finish their defecation diary in the last 2 weeks before randomization. After screening, patients will be randomly assigned to the SA group, NSA group, and NA group according to the ratio of 1:1:1. The block randomization will be used in the trial. An independent statistician who is not involved in the implementation or statistical analysis of this trial will generate the blocked randomization sequence by using the software SAS 9.3.

#### Allocation concealment mechanism

The randomization sequence will be stored by the special randomization sequence manager and will not be available to other participating investigators. The allocation schedule will be using a telephone randomization procedure. The clinical research coordinators will be responsible for requesting randomization.

### Implementation

The blocked randomization sequence will be generated by an independent statistician. The recruiter is responsible for registration. The acupuncturist will assign participants to related interventions.

### Blinding

Patients, outcome assessor and statistician will be blinded to the assignments. During the treatment, the adhesive pads will be glued to the corresponding sterilized acupoints in order to make it impossible for patients to distinguish between the use of single-use sterile needles and blunt-tipped placebo needles. When two or more patients are treated at the same time, they will be screened or assigned to separate treatment rooms in order to refraining from communication. Interventions of patients will not be revealed until the statistical analysis is completed.

Every effort should be made to preserve the blind. Should a medical emergency arise that requires identification of the study acupuncture administered in order to manage the acute situation of the patient, the blind can be broken. The investigator should make every effort to contact the clinical gastroenterologist to discuss the use of rescue medication or other necessary treatments. If blinding is broken ahead of time, it is necessary to record the time, reasons, and executive personnel who break the blindness in advance and notify the inspector as soon as possible. And related patients should not continue to participate in the trial, and the trial data cannot be used for efficacy evaluation analysis, but should still be included in the safety analysis data set. Other available treatments should also be provided to related patients.

### Analytical methods

In this study, statisticians who blind to the group assignment will perform analyses using SPSS 22.0 statistical software. Continuous data will be represented by mean ± standard deviation (M ± SD) or median combined with interquartile range, whereas categorical data will be represented by frequency, constituent ratio, and percentage. Student’s *t* test or Wilcoxon rank-sum test will be used for the comparison of continuous variables, and chi-square test or Wilcoxon rank-sum test will be used for the comparison of categorical variables. *P* < 0.05 is considered to indicate statistical significance. Intention-to-treat (ITT) set will be used in all efficacy analysis, which will consist of all patients who have been randomized into groups, and the missing outcome data will be complemented using last observation carried forward (LOCF) or multiple imputation.

For the primary outcome, covariate analysis will be conducted applying a logistic regression model that includes baseline covariates for both pain score and stool consistency. A per-protocol analysis will be used for primary outcome as sensitivity analysis covering patients who complete ≥ 10 sessions and have no major protocol violations (taking other drugs during the trial, etc.).

### Adverse events

During the trial, adverse events will be monitored and recorded in the form of a specific questionnaire by patients, outcome assessors, and acupuncturists. Based on the potential relationship between the needling procedure and adverse events, acupuncturists and related specialists will categorize all adverse events as treatment-related or not within 24 h of occurrence. Common acupuncture-related adverse events consist of dizziness, subcutaneous hematoma, continuous post-needling pain, local bleeding, itching at the sites of needle insertion, and so on.

### Data management

Before recruiting patients, the case report form (CRF) will be established to input and store data. The outcome evaluator will be responsible for recording the corresponding results of the outcome indicators in the appropriate location in the CRF. While the trial is completed, the data management team will lock the database and the researchers can no longer modify the data. The patients’ relevant information, name, ID card number, and telephone number, will be kept anonymously. Some patients participate in the trial blindly and do not reasonably judge whether their own conditions can complete the content of the test, which will increase the drop-off rate. We have trained researchers to establish a good communication relationship with patients to ensure that patients have a more comprehensive understanding and cooperation of the trial before randomization. For patients who discontinue or deviate from intervention protocols, we will try to get as much trial data as possible by telephone, if the patient is willing to provide it.

Documents of patients’ information will be preserved for at least 5 years after publication. If readers and reviewers have any questions, in the meantime, they can contact the corresponding author for access to the original data. In addition, quality controllers will be independently established in each center to review and interpret the data of trial. They will review the progress of the trial, independently of the investigators, and decide whether the trial needs to be terminated early solely on the basis of adverse events.

### Quality control

Experts in acupuncture, gastroenterology, statistics, and methodology reviewed and revised the trial protocol. Pre-specified standard operating procedures (intervention, details in filling CRF, result evaluation, data management) will be used for the training of related staff. All data modifications can be tracked through CRF. Appropriate communication will be maintained with the patients to strengthen their compliance. In addition, quality controllers will be set up in each branch center to control the quality of the research tasks undertaken by each branch center. Furthermore, the head research center inspector will monitor the trial process and data of each branch research center, when 10% and 90% of patients are included, respectively. If problems are found, the branch research center will be rectified and assessed in strict accordance with the relevant standard operating requirements of this trial. The trial will continue after the branch research center passes the assessment.

### Ethics and dissemination

The study protocol has been approved by the Medical Ethics Committee of Beijing University of Chinese. The randomized controlled trial has obtained the registration number (ChiCTR2000030670) and will be conducted in accordance with the rules of the Declaration of Helsinki. The recruiter in each branch center will be responsible for obtaining the informed consent of patients. Patients will be included only after the details of the study are explained to them and signing informed consent forms. The results of the trial will be published in a peer-reviewed academic journal.

## Discussion

IBS is one of the most common functional gastrointestinal diseases and causes a considerable financial burden for individuals and society [10, 29]. This trial will evaluate the efficacy of SA versus NSA and NA in improving the symptoms of IBS. In this trial, we optimized the study design from the following aspects. First, this trial meets the methodological demand for adequate randomization, allocation concealment, and blinding of patients, outcome assessors, and statisticians. Second, patients will be provided 3 treatment sessions per week in the 4-week treatment phase, giving a total of 12 sessions. Compared with “6 treatment sessions over 3 weeks” in a previous study, the “dose” of acupuncture in our trial is more sufficient, which has been well practiced in clinical practice of China [30].

According to the TCM definition of IBS: “suppressed emotions and concerns would induce a liver qi stagnation, which in turn would lead to an attack of the liver on the spleen and stomach. The resulting weakness of the spleen leads to digestion problems. Furthermore, liver qi stagnation would block qi flow in the meridians, which would induce abdominal pain” [31, 32], the liver is an important organ related to IBS. Most IBS patients are accompanied by abnormalities of mood, such as anxiety, depression, and tension, which is in line with the clinical manifestations of liver dysfunction. The main symptoms of IBS are abdominal pain, constipation, and fatigue, which are common manifestations of spleen deficiency syndrome. Therefore, the additional specific acupoints in this trial were selected in accordance with the TCM treatment principles of invigorating the spleen and regulating the liver.

This study still has some noteworthy limitations. First of all, the acupuncturists cannot be blinded due to the nature of the acupuncture intervention. Second, when patients are unsatisfied with the treatment or cannot tolerate the symptoms of IBS, they may prefer to other treatment, which will increase the drop-out rate. In order to give patients a more comprehensive understanding of the trial, we have trained researchers to establish a good communication relationship with patients. Third, the follow-up of 8 weeks is short, which may affect the outcomes of this trial. We will conduct the large-scale trials with long-termfollow-up of patients.

In conclusion, we hope this trial will provide more reliable evidence and guide the large-scale trial of acupuncture treatment for IBS to be carried out more scientifically and reasonably.

### Trial status

Protocol: version 1.0, 9 March 2020.

The first patient was recruited on 20 July 2019. This pilot study is expected to be finished by the end of March 2021.

## Data Availability

Data sharing is not applicable to this article as no datasets were generated or analyzed during the current study.

## Abbreviations

IBS: Irritable bowel syndrome
SA: Specific acupoints
NSA: Non-specific acupoints
NA: Non-acupoints
IBS-SSS: IBS Symptom Severity Scale
PHQ-9: Patient Health Questionnaire-9 depression scale
IBS-QOL: IBS-Quality of Life scale
IBS-AR: IBS Adequate Relief
BBS: Bristol Stool Score (BBS)
IBS-D: Diarrheal-predominant irritable bowel syndrome
TCM: Traditional Chinese medicine
VAS: Visual analog scale
CRF: Case report form
ITT: Intention-to-treat
LOCF: Last observation carried forward

## References

[1] Mearin F, Lacy BE, Chang L, et al. Bowel Disorders. Gastroenterology. 2016;150(6):1393–407. 10.1053/j.gastro.2016.02.031 27144627

[2] Longstreth GF, Thompson WG, Chey WD (2006). Functional bowel disorders. Gastroenterology..

[3] Canavan C, West J, Card T (2014). The epidemiology of irritable bowel syndrome. Clin Epidemiol.

[4] Lovell RM, Ford AC (2012). Global prevalence of and risk factors for irritable bowel syndrome: a meta-analysis. Clin. Gastroenterol. Hepatol.

[5] Maxion-Bergemann S, Thielecke F, Abel F (2006). Costs of irritable bowel syndrome in the UK and US. Pharmacoeconomics..

[6] Sandler RS, Everhart JE, Donowitz M (2002). The burden of selected digestive diseases in the United States. Gastroenterology..

[7] Gralnek IM, Hays RD, Kilbourne A (2000). The impact of irritable bowel syndrome on health-related quality of life. Gastroenterology..

[8] Chang JY, Talley NJ (2010). Current and emerging therapies in irritable bowel syndrome: from pathophysiology to treatment. Trends Pharmacol Sci.

[9] Spiller R, Aziz Q, Creed F (2007). Guidelines on the irritable bowel syndrome: mechanisms and practical management. Gut..

[10] Peyton L, Greene J (2014). Irritable bowel syndrome: current and emerging treatment options. P&T..

[11] Ruepert L, Quartero AO, de Wit NJ, et al. Bulking agents, antispasmodics and antidepressants for the treatment of irritable bowel syndrome. Cochrane Database Syst Rev. 2011. 10.1002/14651858.CD003460.pub3. 10.1002/14651858.CD003460.pub3 PMC8745618 21833945

[12] Leong SA, Barghout V, Birnbaum HG (2003). The economic consequences of irritable bowel syndrome: a US employer perspective. Arch Intern Med.

[13] Li H, He T, Xu Q (2015). Acupuncture and regulation of gastrointestinal function. World J Gastroenterol.

[14] Ouyang H, Chen JDZ (2004). Review article: therapeutic roles of acupuncture in functional gastrointestinal disorders. Aliment Pharmacol Ther.

[15] Sun JH, Wu XL, Meng YF (2015). Electro-acupuncture decreases 5-HT, CGRP and increases NPY in the brain-gut axis in two rat models of Diarrhea-predominant irritable bowel syndrome(D-IBS). BMC Complement Altern Med.

[16] Liu HP, Zhang YH, Qi DB (2017). Downregulation of the spinal NMDA receptor NR2B subunit during electro-acupuncture relief of chronic visceral hyperalgesia. J Physiol Sci.

[17] Zhu X, Liu Z, Niu W, Wang Y, Zhang A, Qu H, Zhou J, Bai L, Yang Y, Li J (2017). SennaeEffects of electroacupuncture at ST25 and BL25 in a -induced rat model of diarrhoea-predominant irritable bowel syndrome [J]. Acupunct Med.

[18] Ma XP, Tan LY, Yang Y, Wu HG, Jiang B, Liu HR, Yang L (2009). Effect of electro-acupuncture on substance P, its receptor and corticotropin-releasing hormone in rats with irritable bowel syndrome. World J Gastroenterol.

[19] Ohman L, Simrén M (2010). Pathogenesis of IBS: role of inflammation, immunity and neuroimmune interactions. Nat Rev Gastroenterol Hepatol.

[20] Schneider A, Enck P, Streitberger K (2006). Acupuncture treatment in irritable bowel syndrome. Gut..

[21] Streitberger K, Kleinhenz J (1998). Introducing a placebo needle into acupuncture research. Lancet..

[22] Liu BY, Xu HF, Ma R (2014). Effect of blinding with a new pragmatic placebo needle: a randomized controlled crossover study. Medicine (Baltimore).

[23] Francis CY, Morris J, Whorwell PJ (1997). The irritable bowel severity scoring system: a simple method of monitoring irritable bowel syndrome and its progress. Aliment Pharmacol Ther.

[24] Kroenke K, Spitzer RL, Williams JB (2001). The PHQ-9: validity of a brief depression severity measure. J Gen Intern Med.

[25] Patrick DL, Drossman DA, Frederick IO (1998). Quality of life in persons with irritable bowel syndrome: development and validation of a new measure. Dig Dis Sci.

[26] Mangel AW (2006). Personal view: adequate relief as a primary endpoint in irritable bowel syndrome. Aliment Pharmacol Ther.

[27] Leventer SM, Raudibaugh K, Frissora CL (2008). Clinical trial: dextofisopam in the treatment of patients with diarrhoea-predominant or alternating irritable bowel syndrome. Aliment Pharmacol Ther.

[28] Camilleri M, Northcutt AR, Kong S (2000). Efficacy and safety of alosetron in women with irritable bowel syndrome: a randomised, placebo-controlled trial. Lancet..

[29] Weinberg DS, Smalley W, Heidelbaugh JJ (2014). American Gastroenterological Association Institute Guideline on the pharmacological management of irritable bowel syndrome. Gastroenterology..

[30] Lembo AJ, Conboy L, Kelley JM (2009). A treatment trial of acupuncture in IBS patients. Am J Gastroenterol.

[31] Cheng XN (1987). Chinese acupuncture and moxibustion.

[32] Maciocia G (1989). The foundations of Chinese medicine.

