# Correction: Acupuncture for the treatment of diarrheal-predominant irritable bowel syndrome: study protocol for a pilot randomized controlled trial

**DOI:** 10.1186/s13063-022-07016-y

**Published:** 2023-01-16

**Authors:** Ling-Yu Qi, Yu Wang, Li-Qiong Wang, Yan-Fen She, Guang-Xia Shi, Ying Li, Li-Li Chi, Bang-Qi Wu, Jian-Feng Tu, Ying Lin, Fang-Ting Yu, Jing-Wen Yang, Cun-Zhi Liu

**Affiliations:** 1 International Acupuncture and Moxibustion Innovation Institute, School of Acupuncture-Moxibustion and Tuina, Beijing University of Chinese Medicine, Beijing, 100029 China; 2 School of Acupuncture-Moxibustion and Tuina, Hebei University of Chinese Medicine, Shijiazhuang, 050299 China; 3 School of Graduate, Chengdu University of Chinese Medicine, Chengdu, 610075 China; 4 Department of Spleen and Stomach, the Affiliated Hospital of Shandong University of Traditional Chinese Medicine, Jinan, 250011 China; 5 National Acupuncture and Moxibustion Clinical Medical Research Center, the First Teaching Hospital of Tianjin University of Traditional Chinese Medicine, Tianjin, 300193 China

**Correction: Trials 22, 253 (2021)**

**https://doi.org/10.1186/s13063-021-05211-x**

Following publication of the original article [1], a minor error was found in the "Trial status" section:

The time in the sentence of ‘The first patient was recruited on 20 July 2019’ is incorrect, the correct sentence should be: The first patient was recruited on 20 July 2020.

The original paper has been updated.
